# Yaws: A Second (and Maybe Last?) Chance for Eradication

**DOI:** 10.1371/journal.pntd.0000275

**Published:** 2008-08-27

**Authors:** Andrea Rinaldi

**Affiliations:** Case Western Reserve University School of Medicine, United States of America

Some diseases are neglected; others are simply forgotten. Yaws belongs to the second group. One of the first diseases to be targeted for eradication on a global scale, yaws almost disappeared, thanks to a massive treatment program started in the 1950s that eliminated it from many endemic areas. Then something went wrong. The combat line somehow broke down in several points, people focused on other health emergencies, and yaws vanished from the political agenda. Not surprisingly, the disease has now resurged in several countries, where mass treatment has had to be reintroduced.

Although rarely fatal, yaws leaves its indelible mark on many of those touched, horribly corroding their skin and bones. Victims are mostly young children, who end up disabled, stigmatized, and poor. However, despite its visible effects, its amenability for eradication, and all the past efforts made to eliminate it, this bacterial infection is still a menace. The present situation calls for immediate action to wipe yaws out forever. Its history, on the other hand, has much to teach on how eradication campaigns should—and should not—be planned and conducted.

## “Yaws Begins Where the Road Ends”

Yaws (the name is believed to originate from “*yaya*,” which in the indigenous Caribbean language means sore), or framboesia tropica (also known by the local names of *buba*, *pian*, *paru*, and *parangi*) is a chronic, contagious, nonvenereal infection caused by the spirochete *Treponema pallidum* subspecies *pertenue*. A disease of the poor, yaws mainly affects populations living in rural areas of warm and humid subtropical countries, where conditions of overcrowding, poor water supply, and lack of sanitation and hygiene prevail. The original distribution of the infection spanned across Africa, Asia, South America, and Oceania, but past eradication campaigns have strongly reduced the geographic extension and global burden; a few foci resist, however, notably in South-East Asia (Indonesia, Timor-Leste, Papua New Guinea) and Africa (Ghana, Republic of the Congo). Direct skin-to-skin contact is the main route for transmission, together with breaks in the skin caused by injuries, bites, or excoriations [1]. According to older accounts, flies may also play a role as vectors for transmission [2].

**Figure 1:**
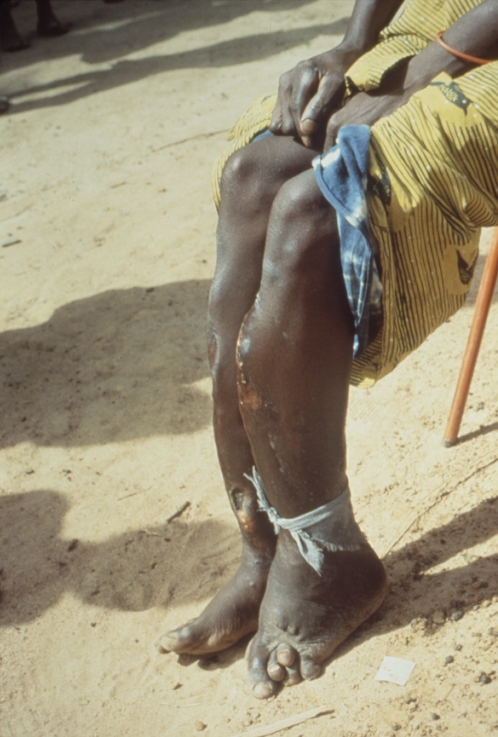
A 45-Year-Old Woman from the Ivory Coast with Tibial “Saber Deformity” as a Result of Late Tertiary Yaws Infection. A saber shin deformity is an abnormality of the tibia characterized by marked anterior bowing of the lower leg. This defect may be seen in some children with congenital syphilis and in patients with yaws. Credit: US Centers for Disease Control and Prevention/Dr. Peter Perine.

Clinical manifestations occur in three distinct phases. At the primary, early stage, a papular “raspberry-like” lesion develops after three to four weeks of incubation at the *Treponema* inoculation site (usually a leg) and ulcerates; this “mother yaw” generally heals spontaneously after a few months, although it can last a year or longer. During the incubation period, spirochetes invade the lymphatics and spread across the body. The secondary stage is characterized by the widespread dissemination of smaller skin lesions that teem with treponemes. Goundou, an osteitis characterized by impressive swellings on each side of the nose and reported sporadically, mostly from West Africa, is recognized as a form of secondary-stage yaws [3]. At the third (late) stage, whose onset can be after a period of latency lasting several years, bone, cartilage, and soft tissue destruction may occur, jointly with severe skin ulceration and painful hyperkeratosis on palms and soles, leading to gross deformities of the legs, skull, nose, palate, and upper jaw, with consequent irreversible disabling and disfiguration (Figures 1–3). This stage is not contagious, and develops in 10% of untreated individuals.

**Figure 2 pntd-0000275-g002:**
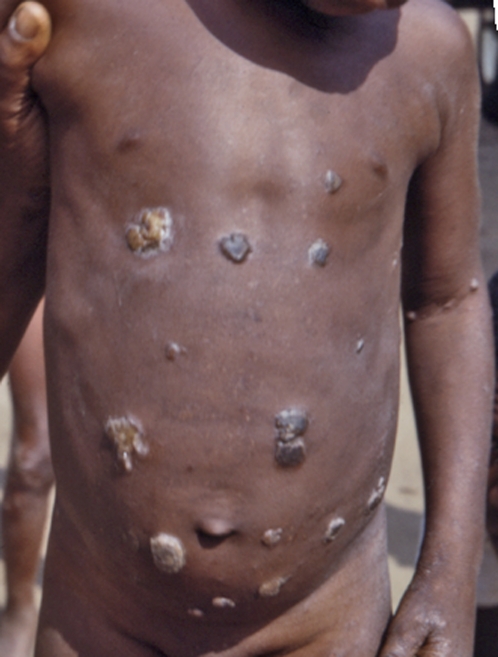
A Nigerian Boy with Yaws (Late 1960s). Yaws causes ulcerative skin lesions, and is found in areas where poor sanitation exists. Yaws was prevalent in some of the relief camps set up during the Nigerian-Biafran war (1967–1970). Credit: US Centers for Disease Control and Prevention/Dr. Lyle Conrad.

**Figure 3:**
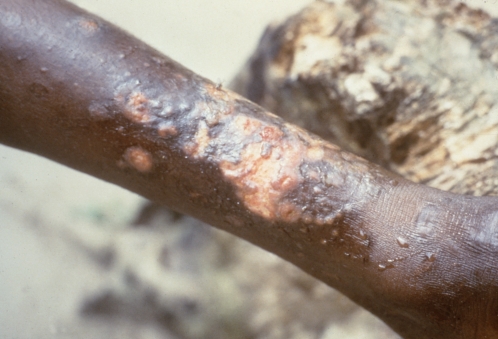
Confluent Papillomata with an Ulceration of the Lower Leg of Several Years Duration in a Yaws Patient. Credit: US Centers for Disease Control and Prevention/Dr. Peter Perine.

Children under 15 years are the main yaws victims, with peak incidence in the six- to ten-year-old age range. Treatment of early-stage yaws is very simple and inexpensive, as it only requires a single intramuscular shot of long-lasting benzathine penicillin that costs as little as 30 US cents. Recurrence due to re-infection or relapse is rare, and only a few penicillin treatment failures have been documented to date, with tetracycline, doxycycline, or erythromycin all being viable alternatives. There is no vaccine, although the availability of the fully sequenced syphilis spirochete genome could lead to important progress in this direction [4],[5]. Thus, prevention relies on early diagnosis and treatment of affected individuals and their contacts, and on implementing personal and group hygiene [1].

## An Old Travel Companion

Treponemal infections form a rather compact cluster. The venereal-transmitted syphilis is by far the best known, owing to its long-lasting presence in Western societies, whereas the nonsexual, tropically distributed yaws, bejel (or endemic syphilis), and pinta have received relatively little attention in medical culture. Different *T. pallidum* subspecies cause syphilis (subsp. *pallidum*), bejel (subsp. *endemicum*), and yaws (subsp. *pertenue*). Pinta, a mild disease characterized by loss of skin pigmentation, is caused by *T. carateum*. All *T. pallidum* subspecies are morphologically identical and cross-react to the same serological tests; only subtle genetic differences have been identified that permit them to be distinguished, leaving open the question of how such a limited variation can translate into the observed differences in pathogenesis [6]. The lack of reliable serological or morphological tests to distinguish *T. pallidum* subspecies obviously limits diagnostic accuracy, which can have serious detrimental effects. For example, treatment for a pregnant woman with burnt-out yaws and one with active syphilis should be different, and assigning the wrong treatment may result in a negative outcome [7]. It is therefore not surprising that an important avenue of current research on treponematoses focuses on the in-depth analysis of the *T. pallidum* genome, in the attempt to identify and decipher determinants of host specificity, pathogenicity, and virulence among the different subspecies (e.g., [8],[9]). Future advances in distinguishing between the subspecies using DNA polymorphisms may lead to serological tests that could be used in the field, facilitating disease control and eradication efforts by permitting the monitoring of its continued impact on populations.

The origin of yaws, as that of the other treponematoses, is still debated. A recent study by a US and Canadian research team, however, has provided fresh insight into the puzzle [10]. These scientists, led by Kristin Harper from Emory University in Atlanta (Georgia, US), used data from a large set of pathogenic *Treponema* strains of disparate geographic origin—including two strains of yaws isolated from remote inhabitants of Guyana in South America—to reconstruct phylogeny and dissemination history. “[E]vidence is consistent with the hypothesis that yaws is an heirloom disease in humans, one caused by a pathogen that infected our anthropoid ancestors and has evolved with our species,” wrote Harper and colleagues [10], claiming that the presence of yaws in wild populations of gorillas, chimpanzees, and baboons further supports this theory, although a more recent cross-species transfer event between humans and nonhuman primates cannot be completely ruled out. According to the derived phylogenetic tree, *T. pallidum* probably first sprouted in the Old World, in the form of nonvenereal infection (yaws), and from there it traveled with humans along their journey to the Middle East and Eastern Europe, changing to endemic syphilis, and then to the Americas, in the form of New World yaws. In the Americas, the causative agent of venereal syphilis, *T. pallidum* subsp. *pallidum*, arose from yaws, as revealed from the genetic analysis of two subsp. *pertenue* strains gathered in Guyana, and was reintroduced back into in the Old World, possibly by the first European explorers. It is also possible that a progenitor of syphilis actually arose in the New World, and thus syphilis itself did not arise until the explorers returned to Europe. These findings, therefore, lend support to the so-called “Columbian theory” of the origin of syphilis, which postulates that Christopher Columbus' crew members unwittingly brought the disease back to Europe when returning from their very first discovery voyage.

Not everyone agrees, however. Commenting on Harper's group's findings, Connie Mulligan and colleagues objected that when the genetic data used to build the phylogenetic tree of *T. pallidum* subspecies are subjected to closer inspection, it appears that no true evolutionary order can be inferred [11]. “Harper et al. have proposed that the syphilis treponeme has evolved more recently in history from New World yaws strains. However, further analysis [by Mulligan and colleagues] is consistent with earlier reports, which do not support an older origin of yaws but a relatively parallel evolution of the three subspecies,” said *Treponema* expert Arturo Centurion-Lara, from the University of Washington at Seattle (Washington, US). “Therefore, we should exercise caution when interpreting biological observations from an evolutionary point of view.” When the rapid disappearance of yaws is considered (see below), archiving strains of yaws and the other nonvenereal treponematoses would allow larger comparative genetic studies, thus helping further research on the history of this disease by providing a data reference tool usable in solving the problem of just how old yaws is and where it came from.

## Eradication: Early Attempts, Failure, Lessons, and New Hopes

Yaws has been the second disease ever to be targeted for eradication (defined as zero incidence and no evidence of transmission determined through surveys among children under five years in areas that were earlier endemic) on a global scale. (The first one was, unsuccessfully, yellow fever, in 1915.) From 1952 to 1964, the Global Yaws Control Programme was launched by the World Health Organization (WHO) in partnership with UNICEF, treating some 300 million people in 46 countries and reducing the global levels of the disease by 95%. Strategy of the early yaws eradication program was based on injection of either benzathine penicillin or procaine penicillin G, and was changed in the campaign's earliest phases to include presumptive therapy for household contacts after it was realized that treatment of overt cases alone produced only a transient reduction in incidence. Treatment coverage varied depending on the prevalence of yaws in a particular area. Where prevalence was less than 5%, only active cases and their contacts were treated; while in areas with levels of endemicity greater than 10%, the entire population was treated [1]. Where prevalence ranged between 5% and 10%, all prepubertal children, adults affected by active disease, and obvious contacts received treatment [1].

When the campaign began—one of the first programs launched by WHO soon after its establishment in 1948—an estimated 50 to 100 million people were afflicted by yaws worldwide. Today's prevalence is unknown, because yaws cases are no longer obligatorily notified to WHO, and many countries where the disease was previously endemic stopped recording them. In the 1990s, estimated global prevalence was at 2.5 million, with 460,000 new cases [12]. “The last information on yaws was in 1990. Since then, there has not been any official notification of the disease. We are currently working with the different regions to identify the remaining endemic countries and foci for targeted elimination efforts,” said Kingsley Asiedu, from the WHO's Department of Control of Neglected Tropical Diseases in Geneva. Currently, about 5,000 new cases are reported annually in South-East Asia, and as many as 26,000 cases were reported in 2005 in Ghana [12]. In Central African countries, yaws has a particularly high prevalence among forest-dwelling Pygmy communities, a fact hinting at their exclusion from government health services [13]. In the Americas, the situation is less clear. Yaws has been eliminated from most of the continent (e.g., [14]), but a survey conducted in 2000 in rural Guyana indicated a prevalence of skin yaws similar to that recorded in 1952 in Haiti, the country with the highest prevalence in the Western Hemisphere before the eradication campaign began [15]. On the other hand, surveys carried out in 2006 and 2007 in endemic yaws territory in Guyana demonstrated no active cases of the disease [10]. “Unfortunately, there are no certain data on the status of yaws in the Americas. The notification of new cases can be significantly underestimated. In the absence of clinic screening and of evaluation of the latent form, it is difficult to consider yaws as eliminated,” said Mariella Anselmi, of the Centro de Epidemiologia Comunitaria y Medicina Tropical (CECOMET) in Esmeralda, Ecuador. “The sporadic cases still recorded in Guyana confirm the persistence of the latent form and thus, given the high contagiousness of lesions, the possibility of epidemic episodes if adequate surveillance is lacking.” In terms of the efficacy of treatment in driving down prevalence of the infection, a study conducted in rural Guyana has shown that treatment of yaws in children less than 14 years of age with an oral penicillin V therapy lasting seven to ten days led to a 71% drop in prevalence. These results demonstrate that a simple but targeted treatment regimen can dramatically reduce the prevalence of yaws in an endemic community and can also effectively treat individuals with active yaws [15].

“The failure to achieve complete eradication was due to multiple reasons, notably the premature integration of yaws control activities into the general health services and the disappearance of support for yaws control,” said Asiedu. Basically, the dedicated vertical programs were dismantled before the final blow could be struck, and the resources and commitment for yaws and its surveillance activities also disappeared. “[Yaws] programs have been deficient in failing to aggressively seek and contain yaws cases and contacts after mass treatment campaigns reduced yaws prevalence to low levels,” wrote Donald Hopkins in 1976, slightly before smallpox global eradication was certified and while yaws was already resurgent [16].

At its eleventh meeting last October, the International Task Force for Disease Eradication (ITFDE)—a group of experts formed at the Carter Center (Atlanta, Georgia, US) in 1988 in the wake of enthusiasm that followed the eradication of smallpox—reviewed the yaws case. ITFDE concluded that “[t]he continued occurrence of yaws and other endemic treponematoses, despite availability of an effective, stable and inexpensive treatment and a simple means of diagnosis in the field, is lamentable testimony to lack of political will, inadequate funding, and persistent weakness in primary health care systems of affected countries” [17]. This view is shared by many, including Jai Narain, Director of the Department of Communicable Diseases of WHO's Regional Office for South-East Asia. “Unfortunately, yaws remains among the most neglected diseases and there is little global attention or focus on this disease, although it primarily affects the most poor and vulnerable sections of the society—the tribal or indigenous people living far away from mainstream,” said Narain. ITFDE lamented that “[t]he current status of knowledge of the extent of yaws is very poor” and suggested that “[t]he World Health Organization should publicize the currently known and unknown status of surveillance for this disease in each of the remaining suspected endemic countries, and encourage mapping and more detailed reporting of surveillance data” [17], thus playing a steering role in addressing this curable and preventable disease.

WHO, it must be stressed, is responding to the appeal. In a consultation round-up held recently at the organization's headquarters in Geneva, experts and officers from the health ministries of those countries where yaws is still a menace agreed on a basic agenda for future action [18]. The agenda includes a rapid assessment of the burden of disease and infection in selected countries; revival of activities to control yaws; and establishment of an elimination program for yaws, directed by WHO in the context of its larger Neglected Tropical Diseases initiative. Elimination of clinical cases (zero cases) supported by active case-finding and serological surveys has been declared as the goal, but no timeline has been set.

Although it is generally agreed that political and financial inertia are the biggest obstacles to interrupting transmission of yaws, some eradication experts point out that biological and medical barriers also exist. For example, although it is usually claimed that humans are the only reservoir of infection, 17% of the members of a wild gorilla population in the Republic of Congo presented typical yaws skin lesions [19]. Yaws may also affect African baboons, and widespread serological testing of wild populations has been performed; genetic analyses of a strain collected from a Guinean baboon shows that it is closely related to human yaws strains [10]. If complete eradication is to be enforced, nonhuman reservoirs need to be cleared as well. Successful treatment campaigns have already been carried out on primates against various infectious diseases; in the case of yaws, affected baboons were darted and treated with penicillin and an additional antibiotic [20]. In parallel, more information is needed on the way pathogens cross-transmit between humans and primate populations, so that suitable strategies to prevent disease spreading and/or resurgence may be developed. As for humans, untreated victims may remain infective for months, favoring the spread of treponemes within populations. A vaccine is still lacking, and so is a reliable test able to distinguish yaws from the various other treponematoses and monitor its impact on populations, as discussed earlier. Furthermore, some researchers noted, the recurrent shortage of benzathine penicillin G in North America due to production problems could make mass treatment difficult ([15]; see also the American Society of Health-System Pharmacists' Web site at http://www.ashp.org/), and the drug's storage requirements may limit its use in tropical areas that lack electricity. “Some of the issues and challenges that have hampered yaws eradication historically include the lack of an effective mechanism for surveillance and case detection, limited political commitment and resources, limited capacity of general health staff to recognize and treat yaws, ensuring drug supply and logistics management, creating community awareness through appropriate advocacy/IEC [Information, Education, and Communication] campaigns and extending the services to remote and hard-to-reach areas,” a recent WHO report remarked [21].

Despite these constraints, however, recent evidence from India suggests that yaws can be effectively controlled, at least at the national level, and even swept out if sufficient energy is applied [22],[23]. “Yaws had been declared to be eliminated from India on Sept 19, 2006 as no case of the disease was reported since 2003. The new target set for yaws eradication from India is year 2008,” said Chandrakant Lahariya, from the Gajara Raja Medical College in Gwalior, India. “The elimination has been achieved based on a two-pronged strategy which primarily consisted of: first, active case finding and treatment of cases and prophylaxis of the household contacts using a single injection of long acting penicillin; second, community mobilisation. The programme used a campaign approach—health workers went village to village every six months looking for yaws cases and offering treatment/prophylaxis to those needing it,” added Narain. “The program is still functioning in the country,” concluded Lahariya. “The biannual independent appraisals of the activities under this program are also done, and the last one was conducted in 2006. The sero-surveillance activities in the selected districts in the children aged less than five years are also being done to ensure that the *T. p. pertenue* is not present in the community,” he said.

As for South-East Asia, attention is now shifting to the last two countries in the region where yaws is still endemic: Indonesia and Timor-Leste [21]. “Indonesia has reported 6,461 cases during 2006 from 17 provinces with majority of cases coming from Nusa Tengarra Timur, Papua, West Papua, Maluku and SE Sulawesi,” said Narain. “These are also the areas which are more remote, relatively inaccessible and less economically well off. In Timor-Leste, the health care workers engaged in immunization campaigns have reported clinical yaws among 1%–2% of children below five, during 2003–2006,” he said. Both these countries are fully committed to yaws eradication and have developed national strategies and operational plans, explained Narain. Many activities such as health care worker training are being carried out. Both countries are also trying to mobilize resources since the constraints of funding remain a major issue, not only for case-finding operations but also for treatment of cases and contacts. “If additional (modest) funds become available, we are hopeful that the target can be achieved within next five years in both Indonesia and Timor-Leste. However this needs the enhanced collaboration and partnership with national and international partners,” said Narain. Lahariya, on his side, thinks that some philanthropic organization should come in and lead the activities of yaws eradication programs in Indonesia and Timor-Leste through sufficiently funded vertical programs. “These are smaller geographical regions, the total fund required for the activities would be small, there is sufficient expertise available in the region: these are the factors to facilitate early yaws eradication from these regions,” said Lahariya.

“We are motivated by India's experience but at the same time, we also know the difficulties the elimination and eradication efforts entail,” said Asiedu. “We have to adapt the strategies to different countries' situations. To make eradication efforts more easier, we may also need to look at new technologies (diagnostics and medicines) to help achieve elimination and eventually eradication. Penicillin is still effective but we also need to research into new antibiotics particularly oral ones.” The insistence on the treatment of subclinical infections (contacts) as well as obvious cases should be rigorously implemented, Asiedu explained. “Treating the sick alone is not sufficient. Today, there are more favorable opportunities to allow integration of yaws activities into the existing general health services.”

## Building on a New Awareness

Besides the general criteria of biological and technical feasibility, costs and benefits, and societal and political considerations, each disease eradication attempt has its own peculiarities, which should be carefully considered in order to achieve success [24]. Yaws, in this regard, is a one-of-a-kind opportunity, because a lot can be learnt from past practical efforts, with less need for speculation. Networking, for example, is now considered a key element for a new strategy to beat the flesh-eating foe. “There is no international network on yaws eradication like you will find with polio and Guinea worm. We need to build the network and develop the necessary partnership for tackling yaws in a sustainable manner,” remarked Asiedu. “Clearly, greater collaboration, networking and resource mobilisation is required in order to achieve yaws eradication globally or even regionally,” agreed Narain. Besides looking at how India achieved success, Asiedu said, activities from other eradication programs like polio, Guinea worm, and leprosy will also be considered. Indeed, collaboration with existing programs could allow resources to be shared and facilitate elimination and surveillance activities. There is also a clear need for increased advocacy and generation of interest, both in the disease itself and in engaging countries.

“What seems most interesting about the renewed effort in South-East Asia is that it is aggressively targeting both cases and their contacts, for which there seems to be evidence that this could overcome the earlier challenges,” said Bruce Aylward, Director of the Global Polio Eradication Initiative (http://www.polioeradication.org/). “The disease is extremely amenable to eradication epidemiologically, technologically, historically and from political commitment point of view. We believe also that efforts on yaws eradication could be an entry point for primary health care for the most marginalised populations,” said Narain. “I strongly believe that yaws can still be eradicated from the world in the next five years,” confirmed Lahariya. “The fortunate thing for yaws is that we don't need whopping amount to realize our efforts. The total amount of money required to achieve yaws eradication would be only a small fraction of what is being spent for polio eradication annually.”

So, after all, the world is being given a new chance to get rid of yaws forever—an accomplishment that could certainly benefit future eradication campaigns with a legacy of positive impetus. “I think that when polio eradication is being proven difficult, achieving yaws eradication will be a big morale booster for the health workforce,” summarized Lahariya. “Ultimately eradication of another disease—take for example that of smallpox in 1978—would by itself be an achievement for public health!” Narain chimed in. The other side of the coin is that a second failure against this vincible enemy could cast discredit and mistrust on other ongoing and future eradication efforts, directed against more pernicious and less vulnerable pathogens. Let's just hope this is not the case.
